# Differential adipokine DNA methylation and gene expression in subcutaneous adipose tissue from adult offspring of women with diabetes in pregnancy

**DOI:** 10.1186/s13148-017-0338-2

**Published:** 2017-04-13

**Authors:** Azadeh Houshmand-Oeregaard, Ninna S. Hansen, Line Hjort, Louise Kelstrup, Christa Broholm, Elisabeth R. Mathiesen, Tine D. Clausen, Peter Damm, Allan Vaag

**Affiliations:** 1 Center for Pregnant Women with Diabetes, Department of Obstetrics, Rigshospitalet, Dept. 7821, Blegdamsvej 9, 2100 Copenhagen, Denmark; 2 Diabetes and Metabolism, Department of Endocrinology, Rigshospitalet, Copenhagen, Denmark; 3 Institute of Clinical Medicine, Faculty of Health and Medical Sciences, University of Copenhagen, Copenhagen, Denmark; 4 Danish Diabetes Academy/Danish PhD School of Molecular Metabolism, Odense, Denmark; 5 Center for Pregnant Women with Diabetes, Department of Endocrinology, Rigshospitalet, Copenhagen, Denmark; 6 Department of Gynecology and Obstetrics, Nordsjaellands Hospital, University of Copenhagen, Hilleroed, Denmark; 7 AstraZeneca, Mölndal, Sweden

**Keywords:** Epigenetics, Methylation, Diabetes, Pregnancy, Gestational diabetes, Fetal programming

## Abstract

**Background:**

Offspring of women with diabetes in pregnancy are at increased risk of type 2 diabetes mellitus (T2DM), potentially mediated by epigenetic mechanisms. The adipokines leptin, adiponectin, and resistin (genes: *LEP*, *ADIPOQ*, *RETN*) play key roles in the pathophysiology of T2DM. We hypothesized that offspring exposed to maternal diabetes exhibit alterations in epigenetic regulation of subcutaneous adipose tissue (SAT) adipokine transcription.

We studied adipokine plasma levels, SAT gene expression, and DNA methylation of *LEP*, *ADIPOQ*, and *RETN* in adult offspring of women with gestational diabetes (O-GDM, *N* = 82) or type 1 diabetes (O-T1DM, *N* = 67) in pregnancy, compared to offspring of women from the background population (O-BP, *N* = 57).

**Results:**

Compared to O-BP, we found elevated plasma leptin and resistin levels in O-T1DM, decreased gene expression of all adipokines in O-GDM, decreased *RETN* expression in O-T1DM, and increased *LEP* and *ADIPOQ* methylation in O-GDM. In multivariate regression analysis, O-GDM remained associated with increased *ADIPOQ* methylation and decreased *ADIPOQ* and *RETN* gene expression and O-T1DM remained associated with decreased *RETN* expression after adjustment for potential confounders and mediators.

**Conclusions:**

In conclusion, offspring of women with diabetes in pregnancy exhibit increased *ADIPOQ* DNA methylation and decreased *ADIPOQ* and *RETN* gene expression in SAT. However, altered methylation and expression levels were not reflected in plasma protein levels, and the functional implications of these findings remain uncertain.

**Electronic supplementary material:**

The online version of this article (doi:10.1186/s13148-017-0338-2) contains supplementary material, which is available to authorized users.

## Background

Early-life exposures may cause persisting changes in offspring metabolism, a concept known as fetal programming [1–3]. Offspring of women with diabetes in pregnancy have an increased risk of obesity, metabolic syndrome, and type 2 diabetes mellitus (T2DM) [1, 2, 4–6]. The risk appears higher than can be explained by genetics [7, 8], implicating a key role for the intrauterine environment. The molecular mechanisms underlying transmission of diabetes risk from mother to offspring are unknown, but may involve modulation of circulating adipokines, which are hormones secreted by adipose tissue. Leptin (gene: *LEP*), adiponectin (gene: *ADIPOQ*), and resistin (gene: *RETN*) are candidate adipokines for investigation of metabolic diseases, as all three are involved in regulation of metabolism, appetite, and insulin sensitivity [9]. High leptin levels are associated with obesity, insulin resistance, and metabolic syndrome, and conversely elevated plasma adiponectin levels are associated with decreased risk of T2DM [10, 11], while associations for resistin are contradictory [11–14].

The changes in offspring metabolism induced by exposure to a detrimental fetal environment are thought to be mediated partly by epigenetic mechanisms, with DNA methylation being the best understood of these mechanisms [15]. Targeted and global epigenetic changes, including changes in methylation of genes encoding adipokines, have been reported in placenta (a central organ in the flux of nutrition from mother to fetus, important for mediating the impact of maternal GDM) and cord blood from newborn offspring in response to prenatal exposure to maternal obesity, hyperglycemia, and GDM [16–27], but the extent to which these changes persist into adulthood is unknown. Studies of the association between maternal glycemia or BMI and offspring adipokine methylation have rendered contradictory results, showing decreased *LEP* and *ADIPOQ* methylation on the fetal side of the placenta with increasing maternal blood glucose concentrations [17, 18] or increased placental *LEP* DNA methylation with exposure to gestational diabetes mellitus (GDM) and maternal obesity [23]. Results on *RETN* methylation are lacking, as are studies of adipokine methylation in adulthood.

The aim of our study was to investigate whether exposure to maternal diabetes causes changes in methylation and gene expression in these adipokines, with corresponding changes in plasma levels, and thereby to test the hypothesis that epigenetic mechanisms controlling adipokine gene expression and secretion are involved in the fetal programming of T2DM.

We measured adipokine plasma levels, gene expression, and DNA methylation in subcutaneous adipose tissue (SAT) in a unique cohort of adult offspring of women with either GDM or type 1 diabetes mellitus (T1DM) in pregnancy, compared to control offspring of women from the background population.

## Methods

### Study design

**Fig. 1:**
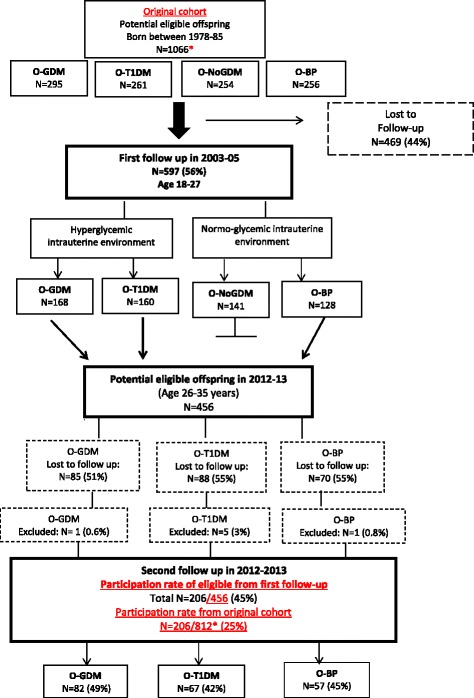
Study design—flowchart of subjects participating and lost to follow-up. *O-GDM* offspring of women with gestational diabetes in pregnancy, *O-NoGDM* offspring of women with risk factors for gestational diabetes but normal glucose tolerance in pregnancy, *O-T1DM* offspring of women with type 1 diabetes in pregnancy, *O-BP* offspring of women from the background population. *In the original cohort, 812 offspring were from the three groups, O-GDM, O-T1DM, and O-BP, due to the fact that O-NoGDM (*n* = 254) were not invited for the second follow-up (1066 − 254 = 812)

The study was an observational follow-up of adult offspring of women with diabetes. Details of the study design, maternal inclusion criteria. and baseline data have been described previously [5, 6, 28]. The original cohort consisted of 1066 adult offspring born between 1978 and 1985 at Rigshospitalet, Denmark. All offspring born to women with either GDM or T1DM, or to women from the background population in this period, were invited (Fig. 1). The participants in this study were between 26 and 35 years old. Of the 597 eligible offspring from the first cross-sectional study, 456 were eligible for participation in this round of follow-up.

Participants belonged to one of three groups depending on exposure to maternal diabetes: offspring of women with diet-treated gestational diabetes (O-GDM, *N* = 82), offspring of women with type 1 diabetes (O-T1DM, *N* = 67), and offspring of women from the background population (O-BP, *N* = 57). Of the 456 eligible offspring from the second follow-up, a total of 45% (49% O-GDM, 42% O-T1DM, and 45% O-BP) participated in this round of follow-up, and there was no inter-group difference in participation rate (*p* = 0.445).

The study was in accordance with the Declaration of Helsinki, approved by the regional ethical committee (ref. nr. H-4-2011-156). All participants received written and oral information and provided written consent before participation.

### Maternal selection criteria and diabetes in pregnancy in 1978–1985

GDM screening was based on risk factors as per clinical procedure during the period of 1978–1985, and a diagnostic 3-h 50-g oral glucose tolerance test (OGTT) was performed in the presence of one or more risk factors and fasting blood glucose ≥4.1 mmol/l [29, 30].

T1DM mothers fulfilled the following criteria: (1) onset of diabetes ≤40 years of age, (2) a classical disease history of hyperglycemic symptoms before diagnosis, and (3) insulin treatment initiated 6 months or less after diagnosis. In the baseline period, HbA1C measurement was not clinical routine, and the procedure for T1DM pregnancies involved hospitalization in the first and third trimesters with measurement of blood glucose seven times a day for 3 days. Mean glucose values were calculated from these 3-day profiles.

Mothers from the background population were unselected women routinely referred for antenatal care and delivery at Rigshospitalet between 1978 and 1985 [6].

### Examination of offspring at follow-up

Participants were recruited and examined between May 2012 and September 2013. They underwent SAT and skeletal muscle biopsies (only the SAT biopsies were used for this study), an OGTT, anthropometric and clinical measurements, and a dual x-ray absorptiometry (DEXA) scan [28].

### Tissue samples

Biopsies were obtained through a small skin incision under local anesthesia from abdominal SAT using a Bergström needle prior to the OGTT. A total of 70–300 mg tissue was obtained, which was immediately frozen and stored at −80 °C until analysis.

SAT biopsies were declined or failed in 11 participants, resulting in a total of *n* = 195 SAT biopsies. Another 21 were lost during the purification process, resulting in a total of *n* = 174 biopsies available for further analysis. Of these, *n* = 161 contained a sufficient amount of tissue and passed quality control and were included in statistical analyses for gene expression. Likewise, only samples containing sufficient amounts of tissue after gene expression analyses as well as those passing strict quality control were included in methylation analyses, resulting in *n* = 123 (*LEP*), *n* = 114 (*ADIPOQ*), and *n* = 135 (*RETN*).

### Outcome variables

The primary outcomes of interest were leptin, adiponectin, and resistin plasma levels, gene expression, and DNA methylation percentage (site-specific and average) in SAT in the three different offspring groups. Furthermore, associations between maternal diabetes status (GDM or T1DM) or maternal blood glucose levels (fasting and 2-h post-OGTT glucose levels in GDM mothers or mean first and third trimester blood glucose levels in T1DM mothers) on the one hand and offspring adipokine gene expression or DNA methylation on the other hand were assessed.

### Exposure variables

Exposure to maternal GDM or T1DM, determined by offspring group, was the primary exposure variable. Maternal pregnancy blood glucose levels (fasting or 2-h post OGTT blood glucose in GDM mothers and mean blood glucose in the first or third trimester in T1DM mothers) were also used as exposures variables in univariate and multivariate regression analyses.

### OGTT, anthropometric measurements, and DEXA scan

After an overnight fast, a 2-h 75-g OGTT was performed and glucose tolerance status assessed according to the 2006 World Health Organization criteria [31]. A DEXA whole-body scanner (GE Medical Systems Lunar Prodigy Advance, Fairfield, CT, USA) was used to assess body composition. BF% was defined as (total fat mass/total body mass) × 100. Weight, height, waist and hip circumference, and blood pressure were measured in duplicates or triplicates, and mean values calculated.

### Venous blood samples

We measured 0-, 30-, and 120-min plasma glucose, fasting HbA1C, triglycerides, HDL and LDL cholesterol, hs-CRP, and adipokine levels. Details regarding blood sampling methods have been described previously [28]. Leptin, adiponectin, and resistin were measured in fasting plasma samples drawn in EDTA-coated vials with enzyme-linked immunoassay using the Meso Scale Discovery (MSD) singleplex platform and analyzed on an MSD MESO QuickPlex SQ 120. The assays were diluted and performed according to the manufacturer’s protocol, and all measurements were performed in duplicates.

### Adipokine gene expression in SAT

Total RNA extraction from SAT biopsies was performed using miRNeasy Mini Kit (Qiagen). Fifty to 90 mg of SAT tissue was used, and a total of 400 ng TNA was used for complementary DNA (cDNA) synthesis. RNA concentrations were measured using a NanoDrop ND 1000 spectrophotometer (Thermo Scientific). The QuantiTect Reverse Transcription Kit (Qiagen) was used for cDNA synthesis. Primers were designed using human-specific databases (Ensembl Genome Browser) and Universal ProbeLibrary (Roche Applied Science, Additional file 1: Table S1), synthesized by DNA Technology, and optimization was performed before use to determine primer working concentrations. *LEP*, *ADIPOQ*, and *RETN* messenger RNA (mRNA) levels were evaluated in duplicates using SYBR Green Master Mix. mRNA expression was measured by reverse transcription quantitative PCR using the ViiA7 Real-Time PCR System (Applied Biosystems) and normalized to the hypoxanthine-guanine phosphoribosyl transferase (HPRT) reference gene. Adipokine gene expression was measured in 78% (161/206) of participants.

### Adipokine DNA methylation in SAT

Genomic DNA was extracted from SAT biopsies using the QIAamp DNA Mini Kit (Qiagen). Twenty to 40 mg SAT tissue was used, and a total of 400 ng DNA was bisulfite converted using the EpiTect Bisulfite Kit (Qiagen). DNA methylation was measured using pyrosequencing. PCR and pyrosequencing primers (Additional file 1: Table S1) were designed using the PyroMark Assay Design 2.0 software, and pyrosequencing of PCR products was performed using the PyroMark Q96 (all Qiagen). We studied CpG sites in the promoter regions and first exon (for *RETN*) of the three genes of interest. *LEP* promoter DNA methylation was measured at CpG sites: −100, −95, −85, −74, −71, −62, and −51 bp upstream from the transcription start site (TSS). These sites were chosen as they are located in a CpG island in the promoter region of the *LEP* gene and have previously been shown to be associated with LEP expression [32]. *ADIPOQ* promoter DNA methylation was measured at CpG sites −112 and −45 bp upstream from the TSS, chosen because they are located in a promoter region shown to be sufficient for basal transcriptional activity [33], and in a site previously found by our group to be differentially methylated after 36 h of fasting in normal-birthweight individuals compared to low-birthweight individuals (unpublished data). *RETN* DNA methylation was measured in the same sites previously shown to be associated with gestational diabetes: −14, −1, +6, +29, relative to the first exon [34].

Adipokine methylation degree was presented as site-specific and average methylation.

Adipokine methylation was measured in 55–66% (114–135/206) of participants.

### Statistical analysis

Statistical analyses were performed using IBM SPSS Statistics version 22. Normally distributed data is presented as mean (SD), while nonparametric data is presented as geometric mean (95% confidence intervals (CI)). Differences between means and proportions were analyzed with independent Student’s *t* tests or chi-square tests, respectively. All comparisons were to the O-BP control group. Forced-entry multiple regression analysis was used to explore the independent association between fetal exposure to diabetes and adipokine gene expression or average DNA methylation. In model 1, we adjusted for potential confounders (maternal prepregnancy BMI, age at delivery, smoking status, family history of diabetes, and offspring gender and age at follow-up), and in model 2, we then added potential mediators (offspring homeostatic model assessment insulin resistance (HOMA-IR) and total body fat % (BF%), HDL cholesterol, waist circumference, and mean systolic and diastolic blood pressure). Gene expression and plasma level values were log transformed in regression analyses in order to meet assumption of homoscedasticity. Correlations were performed using Pearson’s correlation or Spearman’s rank correlation for nonparametric data. Listwise deletion was used in regression analyses; pairwise deletion was used in correlation analyses. A two-sided *p* value <0.05 was considered significant.

## Results

### Characteristics of the study population

Two hundred fifty offspring were lost to follow-up/excluded for various reasons: declined future participation at the first round of follow-up in 2003 (*n* = 19, 7.6%), several unsuccessful attempts at contact by mail/phone (*n* = 94, 37.6%), declined participation (*n* = 88, 35.2%), emigrated (*n* = 13, 5.2%), pregnancy (*n* = 15, 6.0%), illnesses warranting exclusion, including known T1DM or MODY (*n* = 12, 4.8%), traveling, working, or studying abroad (*n* = 2, 0.8%), and a small group lost to follow-up for other reasons (*n* = 7, 2.8%)—leaving a total of 206 participants (45.2%). A previously published dropout analysis of subjects lost to follow-up from the first to the second follow-up found that it was the healthiest offspring who participated in the second follow-up [28].

No difference in the majority of baseline and anthropometric data was found between the exposure groups (O-GDM and O-T1DM) and O-BP, as previously published [28].

Exposed offspring had higher 2-h OGTT glucose values (O-GDM, *p* = 0.016; O-T1DM, *p* = 0.001), and O-GDM demonstrated higher 30-min plasma glucose levels (*p* = 0.006) and borderline higher HbA1C levels (*p* = 0.076) (Table 1).

**Table 1:** Clinical characteristics of the study cohort

	O-GDM	O-T1DM	O-BP	O-GDM vs. O-BP *p* value	O-T1DM vs. O-BP *p* value
*N* (total = 206)	82	67	57		
Maternal characteristics					
Pregestational BMI (kg/m^2^)	24.3 (5.6)	21.7 (1.9)	21.2 (3.5)	**<0.001**	0.301
Maternal age at delivery	30.4 (5.2)	26.4 (4.7)	26.8 (4.6)	**<0.001**	0.645
Maternal smoking status (yes/no)	22/69 (32%)	37/59 (63%)	26/45 (58%)	**<0.006**	0.610
Family history of diabetes (yes vs. no)	26% (21/82)	25% (17/67)	16% (9/57)	0.166	0.191
Offspring anthropometric data					
Age (years)	30.2 (2.1)	30.8 (2.4)	30.8 (2.4)	0.183	0.879
Gender (male)	52% (43/82)	46% (31/67)	46% (26/57)	0.429	0.942
Height (m)	1.76 (0.10)	1.74 (0.10)	1.74 (0.10)	0.481	0.676
Weight (kg)	77.8 (17.4)	78.3 (17.9)	75.3 (16.5)	0.398	0.331
Total body fat (%)	29.8% (0.1)	31.4% (0.1)	28.7% (0.1)	0.428	0.093
BMI (kg/m^2^)	25.2 (5.1)	26.0 (5.9)	24.6 (3.9)	0.493	0.113
Obese (BMI ≥30 kg/m^2^)	15% (12/82)	16% (11/67)	7% (4/57)	0.166	0.110
Waist circumference	85.0 (12.3)	84.3 (11.7)	82.0 (11.2)	0.149	0.265
Mean blood pressure—systolic (mmHg)	117 (9.1)	117 (8.8)	116 (11.9)	0.606	0.626
Mean blood pressure—diastolic (mmHg)	74 (7.43)	71 (9.02)	71.0 (7.28)	0.023	0.859
Results of OGTT^a^					
Fasting plasma glucose (mmol/l)	5.0 (0.7)	4.9 (0.4)	4.9 (0.3)	0.245	0.381
30-min plasma glucose (mmol/l)	8.2 (1.71)	7.8 (1.65)	7.3 (1.6)	**0.006**	0.125
2-h plasma glucose (mmol/l)	6.0 (1.8)	6.3 (1.7)	5.3 (1.2)	**0.016**	**0.001**
HbA1C_DCCT (%)	5.4 (0.3)	5.3 (0.3)	5.3 (0.3)	0.079	0.569
Abnormal glucose tolerance (IFG, IGT, or T2DM)	13% (11/82)	13% (9/67)	5% (3/57)	0.116	0.125
HOMA-IR^b^	1.77 (1.56–2.02)	1.95 (1.71–2.22)	1.72 (1.47–2.02)	0.784	0.222
Plasma samples^b^					
Fasting insulin (pmol/l)	48.9 (43.3–55.2)	54.2 (47.9–61.4)	48.6 (41.9–56.4)	0.953	0.255
Triglycerides (mmol/l)	0.89 (0.81–0.98)	0.84 (0.76–0.93)	1.00 (0.76–1.31)	0.391	0.233
HDL cholesterol (mmol/l)	1.33 (1.26–1.41)	1.44 (1.37–1.52)	1.36 (1.26–1.48)	0.605	0.241

Data is mean (SD) or percentage, unless otherwise indicated. All comparisons are to the O-BP control group. Analysis of differences (means or proportions) between groups was performed by independent samples *t* test or chi-square test, respectively. *p* values <0.05 are in bold

*Abbreviations*: *BMI* body mass index, *HOMA-IR* homeostatic model assessment insulin resistance, *IFG* impaired fasting glucose, *IGT* impaired glucose tolerance, *O-BP* offspring of women from the background population, *OGTT* oral glucose tolerance test, *O-GDM* offspring of women with gestational diabetes, *O-T1DM* offspring of women with type 1 diabetes, *T1DM* type 1 diabetes mellitus, *T2DM* type 2 diabetes mellitus

^a^Based on 2-h 75-g OGTT and evaluated according to the WHO criteria of 2006 [31]

^b^Data is presented as geometric mean and 95% confidence intervals

### Plasma adipokine levels

**Fig. 2:**
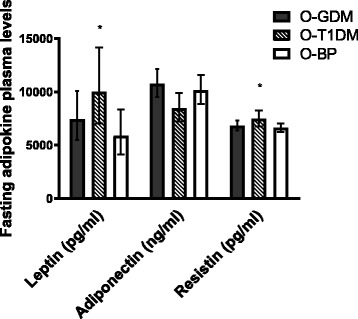
Adipokine plasma levels. Data is geometric mean (95% CI). All values are analyzed by independent samples *t* test after log transformation. All comparisons are to the O-BP control group. Leptin: O-GDM, *N* = 82; O-T1DM, *N* = 66; O-BP, *N* = 57. Adiponectin: O-GDM, *N* = 82; O-T1DM, *N* = 67; O-BP, *N* = 56. Resistin: O-GDM, *N* = 82; O-T1DM, *N* = 67; O-BP, *N* = 57. *p < 0.05. *Black bars*, O-GDM; *striped bars*, O-T1DM; *white bars*, O-BP

Leptin (*p* = 0.034) and resistin (*p* = 0.046) were increased in O-T1DM compared to O-BP with no other significant differences (Fig. 2). No significant difference between groups was found after adjustment for potential confounders and mediators (Additional file 1: Table S2).

### SAT adipokine expression levels

**Fig. 3:**
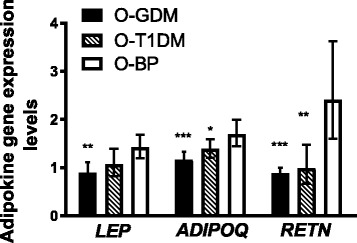
Adipokine gene expression levels in subcutaneous adipose tissue. Data is geometric mean (95% CI). All values are analyzed by independent samples *t* test after log transformation. All comparisons are to the O-BP control group. Levels are relative to the hypoxanthine-guanine phosphoribosyl transferase (HPRT) endogenous control gene. *LEP*: O-GDM, *N* = 58; O-T1DM, *N* = 60; O-BP, *N* = 42. *ADIPOQ* and *RETN*: O-GDM, *N* = 59; O-T1DM, *N* = 60; O-BP, *N* = 42. *p = 0.054, **p < 0.01, ***p < 0.001. Difference in *LEP* expression between O-T1DM and O-BP, *p* = 0.072. *Black bars*, O-GDM; *striped bars*, O-T1DM; *white bars*, O-BP

In crude analyses, the gene expression of all adipokines was significantly lower in O-GDM compared to O-BP (*p* ≤ 0.001). When comparing O-T1DM with O-BP, only *RETN* expression was significantly decreased (*p* = 0.003) (Fig. 3). When adjusting for confounders and mediators, *RETN* expression remained significantly lower in the two glucose-exposed groups (*p* ≤ 0.002) and *ADIPOQ* expression remained lower in O-GDM compared to O-BP (*p* < 0.05) (Additional file 1: Table S3).

### SAT adipokine DNA methylation

**Fig. 4:**
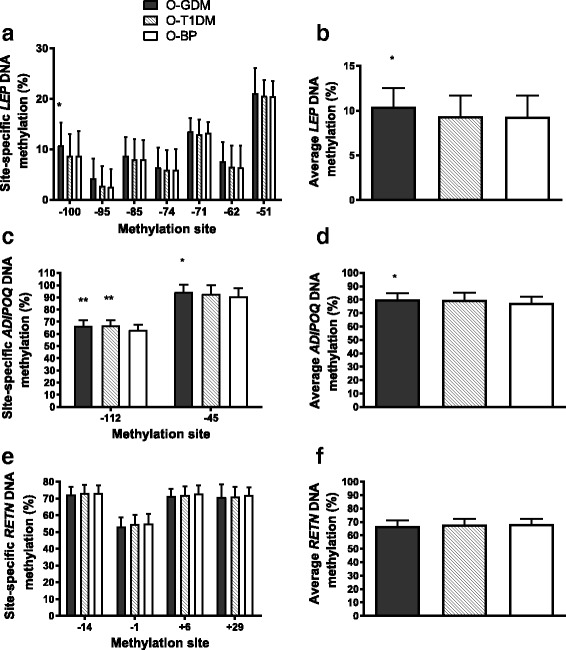
Site-specific and average DNA methylation for **a**, **b** leptin, **c**, **d** adiponectin, and **e**, **f** resistin in subcutaneous adipose tissue. CpG sites are denoted as the number of base pairs upstream (−) or downstream (+) from the transcription start sites. Data is mean ± SD and analyzed using independent samples *t* test. All comparisons are to the O-BP control group. *p < 0.05, **p < 0.01. *Black bars*, O-GDM; *striped bars*, O-T1DM; *white bars*, O-BP

In O-GDM, DNA methylation of *LEP* and *ADIPOQ* was significantly higher in crude analyses (*p* = 0.037 and *p* = 0.022, respectively), while no difference was found in *RETN* DNA methylation. There were no significant differences in adipokine DNA methylation when comparing O-T1DM and O-BP (Fig. 4). After adjustment for confounders and mediators, only *ADIPOQ* methylation remained significantly higher in O-GDM (*p* < 0.05) (Additional file 1: Table S4).

### Correlations between plasma levels, gene expression, DNA methylation, and clinical variables in the whole cohort

#### Leptin

In the cohort as a whole, there was a positive correlation between LEP gene expression and plasma levels but no correlation between LEP DNA methylation levels and gene expression or plasma levels. Offspring clinical markers of metabolic disease correlated positively with leptin plasma levels and gene expression but not with average LEP DNA methylation (Table 2).

**Table 2:** Correlations between leptin plasma levels, gene expression, DNA methylation, and offspring clinical variables in the cohort as a whole (O-GDM, O-T1DM, and O-BP combined)

	Leptin plasma levels	*LEP* gene expression	Average *LEP* DNA methylation
Leptin plasma levels		*0.594 (<0.001)*	0.141 (0.120)
*LEP* gene expression	*0.594 (<0.001)*		−0.001 (0.991)
Average *LEP* DNA methylation	0.141 (0.120)	−0.001 (0.991)	
BMI (kg/m^2^)	*0.472 (<0.001)*	*0.279 (<0.001)*	0.059 (0.519)^a^
Fasting insulin (pmol/l)	*0.540 (<0.001)*	*0.303 (<0.001)*	0.131 (0.163)^a^
Fasting plasma glucose (mmol/l)	0.082 (0.245)	0.121 (0.130)	0.152 (0.094)^a^
120-min plasma glucose (mmol/l)	*0.267 (<0.001)*	*0.317 (<0.001)*	0.173 (0.062)^a^
Total body fat (%)	*0.892 (<0.001)*	*0.564 (<0.001)*	0.126 (0.167)^a^
HOMA-IR	*0.527 (<0.001)*	*0.296 (<0.001)*	0.149 (0.113)^a^
Mean systolic blood pressure (mmHg)	−0.138 (0.050)	−0.127 (0.112)	0.032 (0.725)^a^
Mean diastolic blood pressure (mmHg)	*0.368 (<0.001)*	*0.187 (0.019)*	0.134 (0.138)^a^
Waist circumference (cm)	*0.206 (0.003)*	0.149 (0.061)	0.096 (0.289)^a^
HDL cholesterol (mmol/l)	0.071 (0.316)	−0.008 (0.919)	−0.133 (0.141)^a^

Data is Spearman’s rank coefficient *R* (*p* value) unless otherwise indicated. *p* values <0.05 are in italics

*O-GDM* offspring of women with gestational diabetes, *O-T1DM* offspring of women with type 1 diabetes, *O-BP* offspring of women from the background population

^a^Pearson’s correlation coefficient

#### Adiponectin

There was a positive correlation between *ADIPOQ* gene expression and plasma levels, and a negative correlation between *ADIPOQ* methylation and gene expression, but no correlation between *ADIPOQ* DNA methylation and plasma levels. Offspring clinical markers of metabolic disease correlated negatively with adiponectin plasma levels and gene expression, but positively with ADI DNA methylation (Table 3).

**Table 3:** Correlations between adiponectin plasma levels, gene expression, DNA methylation, and offspring clinical variables in the cohort as a whole (O-GDM, O-T1DM, and O-BP combined)

	Adiponectin plasma levels	*ADIPOQ* gene expression	Average *ADIPOQ* DNA methylation
Adiponectin plasma levels		*0.169 (0.032)*	−0.084 (0.376)
*ADIPOQ* gene expression	*0.169 (0.032)*		−*0.266 (0.013)*
Average *ADIPOQ* DNA methylation	−0.084 (0.376)	−*0.266 (0.013)*	
BMI (kg/m^2^)	−*0.195 (0.005)*	−*0.417 (<0.001)*	*0.359 (<0.001)* ^a^
Fasting insulin (pmol/l)	−*0.281 (<0.001)*	−*0.341 (<0.001)*	*0.318 (0.001)* ^a^
Fasting plasma glucose (mmol/l)	−*0.145 (0.039)*	−*0.163 (0.039)*	*0.195 (0.038)* ^a^
120-min plasma glucose (mmol/l)	−*0.141 (0.049)*	−0.063 (0.441)	*0.219 (0.022)* ^a^
Total body fat (%)	−0.078 (0.268)	−*0.164 (0.038)*	*0.212 (0.024)* ^a^
HOMA-IR	−*0.289 (<0.001)*	−*0.360 (<0.001)*	*0.344(<0.001)* ^a^
Mean systolic blood pressure (mmHg)	−*0.195 (0.005)*	−*0.274 (<0.001)*	0.175 (0.062)^a^
Mean diastolic blood pressure (mmHg)	−*0.139 (0.048)*	−*0.351 (<0.001)*	0.148 (0.115)^a^
Waist circumference (cm)	−*0.239 (0.001)*	−*0.439 (<0.001)*	*0.447 (<0.001)* ^a^
HDL cholesterol (mmol/l)	*0.240 (0.001)*	*0.356 (<0.001)*	−*0.286 (0.002)* ^a^

Data is Spearman’s rank coefficient *R* (*p* value) unless otherwise indicated. *p* values <0.05 are in italics

*O-GDM* offspring of women with gestational diabetes, *O-T1DM* offspring of women with type 1 diabetes, *O-BP* offspring of women from the background population

^a^Pearson’s correlation coefficient

#### Resistin

For resistin, there were no correlations between plasma levels, gene expression, or DNA methylation. There was a positive correlation between resistin plasma levels and a negative correlation between *RETN* DNA methylation and offspring clinical markers of metabolic disease. There were no significant correlations with *RETN* gene expression (Table 4).

**Table 4:** Correlations between resistin plasma levels, gene expression, DNA methylation, and offspring clinical variables in the cohort as a whole (O-GDM, O-T1DM, and O-BP combined)

	Resistin plasma levels	*RETN* gene expression	Average *RETN* DNA methylation
Resistin plasma levels		0.070 (0.380)	−0.162 (0.061)
*RETN* gene expression	0.070 (0.380)		0.109 (0.270)
Average *RETN* DNA methylation	−0.162 (0.061)	0.109 (0.270)	
BMI (kg/m^2^)	*0.177 (0.011)*	−0.110 (0.165)	*−0.270 (0.002)* ^a^
Fasting insulin (pmol/l)	*0.198 (0.007)*	0.003 (0.970)	*−0.327 (<0.001)* ^a^
Fasting plasma glucose (mmol/l)	*0.154 (0.027)*	−0.051 (0.523)	*−0.183 (0.033)* ^a^
120-min plasma glucose (mmol/l)	0.022 (0.762)	0.002 (0.977)	−0.089 (0.318)^a^
Total body fat (%)	*0.230 (0.001)*	−0.044 (0.577)	*−0.320 (<0.001)* ^a^
HOMA-IR	*0.224 (0.002)*	−0.012 (0.883)	*−0.344 (<0.001)* ^a^
Mean systolic blood pressure (mmHg)	0.079 (0.262)	−0.116 (0.144)	−0.051 (0.555)^a^
Mean diastolic blood pressure (mmHg)	*0.212 (0.002)*	*−0.183 (0.021)*	*−0.233 (0.007)* ^a^
Waist circumference (cm)	0.058 (0.409)	−0.131 (0.099)	*−0.260 (0.002)* ^a^
HDL cholesterol (mmol/l)	−0.017 (0.813)	0.016 (0.844)	0.096 (0.268)^a^

Data is Spearman’s rank coefficient *R* (*p* value) unless otherwise indicated. *p* values <0.05 are in italics

*O-GDM* offspring of women with gestational diabetes, *O-T1DM* offspring of women with type 1 diabetes, *O-BP* offspring of women from the background population

^a^Pearson’s correlation coefficient

When correlations between clinical variables and adipokine plasma levels as well as gene expression and average DNA methylation in SAT were explored in the different subgroups, the same patterns were found, although associations were not all statistically significant (Additional file 1: Tables S5–S7).

### Evaluating potential associations between maternal blood glucose levels during pregnancy and offspring adipokine plasma levels, gene expression, and DNA methylation

In univariate analyses, maternal fasting/2-h blood glucose levels (for O-GDM) and maternal mean blood glucose levels in the first/third trimester (for O-T1DM) were not significantly associated with adipokine plasma or gene expression levels. In multivariate regression analyses, *ADIPOQ* expression was borderline significantly associated with maternal fasting blood glucose levels in model 1, and this association became significant in model 2 (*p* = 0.040). *ADIPOQ* expression levels were significantly positively associated with mean maternal glucose levels in the first trimester (*p* = 0.016) in model 1, but this association was no longer significant in model 2.

Univariate analyses between maternal blood glucose levels and offspring adipokine methylation showed a significant positive association between maternal fasting blood glucose levels and *LEP* and *ADIPOQ* methylation (*LEP*, *p* = 0.022; *ADIPOQ*, *p* = 0.050) for O-GDM. These associations were no longer significant after adjustment for potential confounders and mediators.

## Discussion

We found no differences in plasma adipokine levels, decreased adipokine gene expression levels, and increased DNA methylation of *LEP* and *ADIPOQ* in O-GDM compared to O-BP. In O-T1DM, we found increased plasma leptin and resistin levels, decreased *ADIPOQ* and *RETN* gene expression levels, and no difference in average DNA methylation levels compared to O-BP.

The decreased *ADIPOQ* expression and increased average *ADIPOQ* methylation in O-GDM remained significant after adjustment for potential confounders and mediators, implying a role for hyperglycemia in pregnancy in causing these changes. Altered methylation or gene expression levels could lead to changes in paracrine adipokine functions that are not reflected by altered plasma levels alone, which could explain the lack of difference in adiponectin plasma levels in O-GDM despite altered *ADIPOQ* expression and DNA methylation. Thus, the increased *ADIPOQ* methylation levels in O-GDM may lead to changes that we are not aware of. Maternal GDM and T1DM also both remained significantly negatively associated with *RETN* gene expression, but not with *RETN* methylation, implying that methylation of the sites studied here is not responsible for mediating the changes in *RETN* expression associated with exposure to maternal diabetes. *ADIPOQ* methylation and gene expression both remained associated with maternal GDM even after adjustment for potential confounders and mediators.

The fact that SAT *LEP* DNA methylation was not consistently associated with maternal or offspring factors is to some extent in contrast to other results obtained in placenta or cord blood samples of newborns [17, 22–24, 35]. The explanation for the different findings may besides age differences be due to differences in the methylation sites and tissues studied. One study found that 19–28% of variability in offspring *LEP* methylation was explained by maternal hyperglycemia, leaving the quantitatively largest portion to be explained by other factors [17]. Maternal fasting blood glucose levels became significantly associated with *ADIPOQ* gene expression after adjustment for confounders and mediators, but the sample size in these analyses was quite low (*n* = 45), and the findings could be due to a type 1 error.

We found increased leptin and resistin plasma levels in O-T1DM compared to O-BP, but especially for leptin, these changes disappeared after adjustment for potential confounders and mediators. A previous study found hyperleptinemia in 6–13-year-old offspring of women with GDM or T1DM [36]. These differences were attenuated after adjustment for offspring BMI, similar to our findings, indicating that plasma leptin levels are determined largely by body composition. However, the lack of consistent directional changes in plasma leptin levels versus adipose tissue gene expression and methylation levels is complicated by the development of leptin resistance and/or impaired leptin clearance along with adiposity development. Thus, it cannot be excluded that obesity development in O-GDM and O-T1DM may have been initiated by subtle impairments of adipose tissue leptin production due to increased *LEP* DNA methylation causing impaired appetite suppression, adiposity, and eventually a new balanced/compensated metabolic state with normal or increased plasma leptin levels in the face of decreased tissue gene expression levels. Furthermore, adjusting for potential mediators bears a risk of over-adjustment.

In our unique study material, the correlations between *LEP* and *ADIPOQ* expression and plasma levels on the one hand and offspring clinical variables on the other hand in the cohort as a whole are meaningful from a clinical point of view and in line with previous reports [37, 38]. Resistin was named due to its ability to modulate insulin resistance, but studies of resistin associations with clinical parameters of metabolic disease have shown both positive and negative correlations with obesity, insulin resistance, and other metabolic parameters [12–14]. Our results clearly show a positive correlation between resistin plasma levels and clinical parameters of metabolic disease.

When examining the offspring groups separately, the same pattern of associations between plasma levels, gene expression, methylation, and metabolic parameters were found as in the whole cohort, although attenuated.

T1DM usually presents with higher glucose values than GDM, evidenced by the higher proportion of O-T1DM born large for gestational age [5, 28], but differences in gene expression and methylation were generally greater for O-GDM vs. O-BP, indicating that factors other than intrauterine hyperglycemia may play a role in mediating the observed differences. Maternal GDM is a precursor of T2DM, and offspring of GDM women are likely to be more genetically predisposed to T2DM, potentially accounting for the greater differences in gene expression. Moreover, maternal prepregnancy BMI may play a role, as mothers with GDM are generally heavier than mothers with T1DM, which was also the case in our cohort [28]. Finally, postnatal lifestyle factors may also play a role.

### Study strengths and limitations

We are unaware of other studies with epigenetic, transcriptomic, and proteomic data from adult offspring exposed to maternal diabetes, and the availability of this information is one of the strengths of our study. Furthermore, our study is unique due to the availability of metabolically important target tissue in the relatively large sample size. Studies analyzing adipokine methylation, expression, or plasma levels in offspring exposed to intrauterine hyperglycemia have mainly been performed in placental tissues or umbilical cord blood from newborns [16, 17, 39–41], or young children [36, 42], but tissue specificity of epigenetic marks makes it difficult to extrapolate these results to other tissues. The availability of maternal blood glucose values constitutes another strength of this study, but more sophisticated measures of maternal glycemia such as HbA1C or home blood glucose measurements would have been more precise indicators of maternal glycemia.

Study limitations, addressed previously [28], include selection bias as well as residual confounding. Twenty-five percent of offspring from the original cohort participated in the second round of follow-up on which this study is based, and those lost to follow-up included the least healthy subjects diagnosed with prediabetes, metabolic syndrome, or T2DM already during the first follow-up, as previously shown [28]. Although this is an important cause of selection bias, it will tend to push towards an underestimation of our results.

As mentioned in the “Methods” section, gene expression and methylation analyses were not measured in all subjects. However, since different subsets were available for the different analyses, and as such there was no systematic selection in the population undergoing these analyses, we do not believe this biased our results significantly. Due to our a priori knowledge of adipose tissue adipokines playing a major role in the development of adiposity and associated diseases including T2DM, we used a target candidate approach to characterize adipose tissue DNA methylation, gene expression levels, and circulating levels of the three major adipokines. Compared with the alternative approach of applying random DNA methylation or gene expression array approaches, we avoided complicated statistical and bioinformatical analyses with corrections for multiple comparisons. Thereby, we provided compelling proof of principle of potentially functionally important epigenetic and transcriptional gene regulation changes in adipose tissue biopsies from offspring of women with diabetes in pregnancy. Indeed, these findings pave the way for additional large-scale epigenetic and transcriptional omics studies in this unique cohort to explore additional epigenetic mechanisms involved in programming of metabolic disease.

Although the specific genes studied here are known to play an important role in the pathophysiology of metabolic disease [9], other cytokines, signal molecules, and methylation sites are likely to be involved in the fetal programming of metabolic disease. Finally, other epigenetic mechanisms besides methylation (e.g., miRNAs) could also be involved in mediating adipokine expression and function. Thus, the current study does not provide a complete view of the metabolic pathways potentially affected by exposure to maternal diabetes.

The authors wish to emphasize that the current data were obtained in biopsies of predominantly mature adipose tissue cells influenced to some unknown extent by the ambient in vivo metabolic, endocrine and paracrine environment. This is in contrast to another recent paper including isolated preadipocytes in a subgroup from the same cohort of offspring of women with and without diabetes in pregnancy cultured during standardized and controlled in vitro conditions [43], explaining the differential epigenetic and transcriptional findings in the two papers.

## Conclusion

Exposure to maternal GDM was associated with increased *ADIPOQ* methylation and decreased *ADIPOQ* and *RETN* gene expression after adjustment for potential confounders and mediators. Especially for *ADIPOQ* methylation and expression, our findings support our original hypothesis that epigenetic mechanisms controlling adipokine gene expression may be involved in fetal programming of T2DM. The decreased adipokine gene expression levels were not reflected in plasma protein levels, and further studies are needed to understand the functional implications of these findings.

## Additional files

Additional file 1: Figure S1. Adipokine DNA methylation sites studied. **Table S1.** Primers for RT-qPCR and DNA methylation. **Table S2.** Adipokine plasma levels. **Table S3.** Adipokine gene expression levels in subcutaneous adipose tissue. **Table S4.**Average Adipokine DNA methylation levels in subcutaneous adipose tissue. **Table S5.** Correlations between leptin plasma levels, gene expression, DNA methylation and clinical variables by offspring group. **Table S6.** Correlations between adiponectin plasma levels, gene expression, DNA methylation and clinical variables by offspring group. **Table S7.** Correlations between resistin plasma levels, gene expression, DNA methylation and clinical variables by offspring group. (DOCX 63 kb)

## Abbreviations

*ADIPOQ*: Adiponectin gene
BF%: Total body fat percentage: (total fat mass/total body mass) × 100
HDL: High-density lipoprotein
HOMA-IR: Homeostatic model assessment insulin resistance
hs-CRP: High-sensitivity C-reactive protein
IFG: Impaired fasting glucose
IGT: Impaired glucose tolerance
LDL: Low-density lipoprotein
*LEP*: Leptin gene
mRNA: Messenger RNA
O-BP: Offspring of women from the background population (control group)
O-GDM: Offspring of women with gestational diabetes
OGTT: Oral glucose tolerance test
O-NoGDM: Offspring of women with risk factors for GDM but normal OGTT in pregnancy
O-T1DM: Offspring of women with type 1 diabetes
*RETN*: Resistin gene
SAT: Subcutaneous adipose tissue
T1DM: Type 1 diabetes mellitus
T2DM: Type 2 diabetes mellitus
