# Supplementary material for: Differential adipokine DNA methylation and gene expression in subcutaneous adipose tissue from adult offspring of women with diabetes in pregnancy
Source: Clin Epigenetics. 2017 Apr 13;9:37. doi: 10.1186/s13148-017-0338-2 (PMC5390345; doi:10.1186/s13148-017-0338-2)
Supplement: Supplementary file 1 — Adipokine DNA methylation sites studied. Table S1. Primers for RT-qPCR and DNA methylation. Table S2. Adipokine plasma levels. Table S3. Adipokine gene expression levels in subcutaneous adipose tissue. Table S4.Average Adipokine DNA methylation levels in subcutaneous adipose tissue. Table S5. Correlations between leptin plasma levels, gene expression, DNA methylation and clinical variables by offspring group. Table S6. Correlations between adiponectin plasma levels, gene expression, DNA methylation and clinical variables by offspring group. Table S7. Correlations between resistin plasma levels, gene expression, DNA methylation and clinical variables by offspring group. (DOCX 63 kb) [file 13148_2017_338_MOESM1_ESM.docx]

**Additional file 1**

**Figure S1 - Adipokine DNA methylation sites studied**


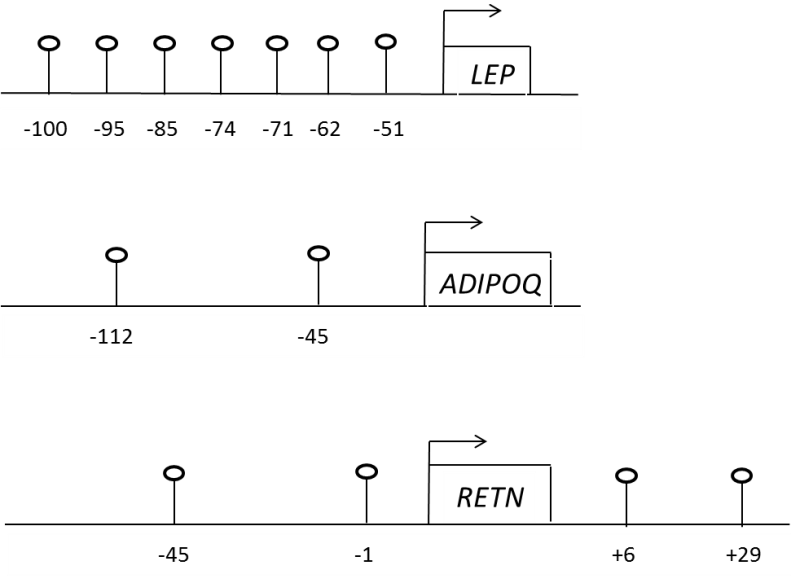


CpG sites are denoted as number of base pairs upstream (-) or downstream(+) from the transcription start site.

Abbreviations: *LEP*, leptin gene; *ADIPOQ*, adiponectin gene; *RETN*, resistin gene.

**Table S1: Primers for RT-qPCR and DNA methylation**

| **Gene** | **Primer Sequence (RNA), 5′→3′** |
| --- | --- |
| **Leptin** | Forward: GAACCCTGTGCGGATTCTTGT  Reverse: TCCATCTTGGATAAGGTCAGGAT |
|  |  |
| **Adiponectin** | Forward: ATGGCCCCTGCACTACTCTA  Reverse: CAGGGATGAGTTCGGCACTT |
|  |  |
| **Resistin** | Forward: TGCAGGATGAAAGCTCTCTGT  Reverse: GGGTCTTGCTAGACACCAACA |
|  |  |
| **Gene Primers for DNA Methylation 5′→3′** | |
| **Leptin** | Forward: AGTTATTTTTAAATTTTTGGGAGGTATT |
|  | Reverse: biotin-ACTACTAACCCTAAACCCCCAATATAC |
|  | Sequencing: ATTTTTGGGAGGTATTTAAG |
| **Adiponectin** | Forward: GTGGGTAATTGTTAGGGATATGT |
|  | Reverse: biotin-AAAAAATAACCCAACCTCAACAAC |
|  | Sequencing: GTAATTGTTAGGGATATGTG |
| **Resistin** | Forward: 5´-AGGGATTTATTAGTTAAGTTAGG-3´  Reverse: 5´-biotin-ACAAACCCAAAAACAACTATCAC-3´  Sequencing: 5´-AGAGGTTTTAAAGAAAGAGT-3´ |

**Table S2: Adipokine plasma levels**

|  | **O-GDM** | | **O-T1D** | | **O-BP** | | **O-GDM vs O-BP p-value** | **O-T1DM vs O-BP p-value** |
| --- | --- | --- | --- | --- | --- | --- | --- | --- |
|  | **N** | **Geometric mean (95% CI)** | **N** | **Geometric mean (95% CI)** | **N** | **Geometric mean (95% CI)** |  |  |
| **Leptin (pg/ml)** | 82 | 7457  (5502-10107) | 66 | 10011  (7074-14168) | 57 | 5891  (4159-8343) | 0.315  Model 1: 0.318  Model 2: 0.915 | **0.034**  Model 1: **0.028**  Model 2: 0.120 |
| **Adiponectin (ng/ml)** | 82 | 10760  (9519-  12162) | 67 | 8477  (7248  -9915) | 56 | 10141  (8851-  11617) | 0.526  Model 1: 0.722  P-value Model 2: 0.819 | 0.093  Model 1: 0.154  P-value Model 2: 0.242 |
| **Resistin (pg/ml)** | 82 | 6830  (6386-  7306) | 67 | 7474  (6769-  8253) | 57 | 6636  (6226-  7073) | 0.536  Model 1: 0.917  Model 2: 0.645 | **0.046**  Model 1: 0.225  Model 2: 0.371 |

All comparisons are to O-BP control group, performed by independent samples t-test. Values are rounded to the nearest whole number.

Abbreviations: O-GDM: offspring of women with gestational diabetes; O-T1D: offspring of women with type 1 diabetes; O-BP: offspring of women from the background population. Model 1: Maternal pre-pregnancy BMI, age at delivery, smoking status, family history of diabetes, offspring gender and offspring age at follow-up

Model 2: Model 1 with addition of the following mediators: Offspring HOMA-IR, total body fat % , HDL cholesterol, waist circumference, mean systolic and diastolic blood pressure.

**Table S3: Adipokine gene expression levels in subcutaneous adipose tissue**

|  | **O-GDM** | | **O-T1D** | | **O-BP** | | **O-GDM vs O-BP p-value** | **O-T1DM vs O-BP p-value** |
| --- | --- | --- | --- | --- | --- | --- | --- | --- |
|  | **N** | **Geometric mean**  **(95% CI)** | **N** | **Geometric mean (95% CI)** | **N** | **Geometric mean (95% CI)** |  |  |
| **Leptin** | 58 | 0.89 (0.71-1.11) | 60 | 1.07 (0.82-1.40) | 42 | 1.43 (1.2-1.69) | **0.001**  Model 1: 0.164  Model 2: 0.095 | 0.072  Model 1: 0.297  Model 2: 0.165 |
| **Adiponectin** | 59 | 1.17 (1.03-1.33) | 60 | 1.39 (1.21-1.59) | 42 | 1.70 (1.45-1.99) | **<0.001**  Model 1: **0.010**  Model 2: **0.030** | 0.054  Model 1: 0.287  Model 2: 0.453 |
| **Resistin** | 59 | 0.88(0.76-1.00) | 60 | 0.99 (0.67-1.48) | 42 | 2.41 (1.60-3.62) | **<0.001**  Model 1: **<0.001**  Model 2: **<0.001** | **0.003**  Model 1: **0.002**  Model 2: **0.001** |

All comparisons are to O-BP control group, performed by independent samples t-test.

Abbreviations: O-GDM: offspring of women with gestational diabetes; O-T1D: offspring of women with type 1 diabetes; O-BP: offspring of women from the background population.

Gene expression levels are relative to HPRT (hypoxanthine-guanine phosphoribosyltransferase) endogenous control gene.

Model 1: Maternal pre-pregnancy BMI, age at delivery, smoking status, family history of diabetes, offspring gender and offspring age at follow-up

Model 2: Model 1 with addition of offspring HOMA-IR, total body fat % , HDL cholesterol, waist circumference, mean systolic and diastolic blood pressure as mediators.

**Table S4: Average adipokine methylation levels in subcutaneous adipose tissue**

|  | **O-GDM** | | **O-T1D** | | **O-BP** | | **O-GDM vs O-BP p-value** | **O-T1DM vs O-BP p-value** |
| --- | --- | --- | --- | --- | --- | --- | --- | --- |
|  | **N** | **Mean**  **(95% CI)** | **N** | **Mean (95% CI)** | **N** | **Mean (95% CI)** |  |  |
| **Leptin (%)** | 47 | 10.4 (9.8-11.0) | 45 | 9.4 (8.7-10.1) | 31 | 9.3 (8.5-10.2) | **0.037**  Model 1: 0.251  Model 2: 0.129 | 0.958  Model 1: 0.403  Model 2: 0.866 |
| **Adiponectin (%)** | 43 | 80.2 (78.7-81.6) | 43 | 80.0 (78.3-81.5) | 28 | 77.5 (75.7-79.3) | **0.022**  Model 1: **0.008**  Model 2: **0.012** | 0.056  Model 1: 0.101  Model 2: 0.083 |
| **Resistin (%)** | 52 | 66.9 (65.7-68.1) | 47 | 67.9 (66.6-69.2) | 36 | 68.3 (68.9-69.7) | 0.141  Model 1: 0.988  Model 2: 0.657 | 0.674  Model 1: 0.199  Model 2: 0.191 |

All comparisons are to O-BP control group, performed by independent samples t-test.

Abbreviations: O-GDM: offspring of women with gestational diabetes; O-T1D: offspring of women with type 1 diabetes; O-BP: offspring of women from the background population.

Model 1: Maternal pre-pregnancy BMI, age at delivery, smoking status, family history of diabetes, offspring gender and offspring age at follow-up

Model 2: Model 1 with addition of the following mediators: Offspring HOMA-IR, total body fat % , HDL cholesterol, waist circumference, mean systolic and diastolic blood pressure.

**Table S5: Correlations between leptin plasma levels, gene expression, DNA methylation and clinical variables by offspring group**

| **Leptin** | | | | | | | | | |
| --- | --- | --- | --- | --- | --- | --- | --- | --- | --- |
| **Offspring data** | **Leptin plasma levels** | | | ***LEP* gene expression** | | | **Average *LEP* DNA methylation** | | |
|  | **O-GDM** | **O-T1DM** | **O-BP** | **O-GDM** | **O-T1DM** | **O-BP** | **O-GDM** | **O-T1DM** | **O-BP** |
| **Plasma levels** | **-** | **-** | **-** | **0.574 (<0.001)** | **0.762 (<0.001)** | **0.433 (0.004)** | **0.316 (0.031)** | -0.054 (0.730) | 0.145 (0.437) |
| **Gene expression** | **0.574 (<0.001)** | **0.762 (<0.001)** | **0.433 (0.004)** | **-** | **-** | **-** | **-0.001 (0.997)** | **0.042 (0.800)** | **0.211 (0.312)** |
| **Average DNA methylation** | **0.316 (0.031)** | -0.054 (0.730) | 0.145 (0.437) | -0.001 (0.997) | 0.042 (0.800) | 0.211 (0.312) | **-** | **-** | **-** |
| **BMI (kg/m^2^)** | **0.528 (<0.001)** | **0.513 (<0.001)** | **0.330 (0.012)** | 0.188 (0.157) | **0.383 (0.003)** | **0.314 (0.043)** | 0.086 (0.565)^a^ | 0.073 (0.633)^a^ | 0.110 (0.556)^a^ |
| **Fasting insulin** | **0.524 (<0.001)** | **0.603 (<0.001)** | **0.512 (<0.001)** | 0.165 (0.242) | **0.448 (0.001)** | 0.182 (0.281) | **0.479 (0.001)**^a^ | -0.132 (0.418)^a^ | 0.062 (0.746)^a^ |
| **Fasting plasma glucose** | 0.130 (0.244) | 0.111 (0.375) | -0.015 (0.911) | 0.092 (0.492) | **0.262 (0.044)** | 0.025 (0.876) | 0.183 (0.218)^a^ | 0.085 (0.578)^a^ | 0.207 (0.263)^a^ |
| **120-min plasma glucose** | **0.334 (0.003)** | 0.230 (0.065) | 0.080 (0.560) | **0.453 (0.001)** | **0.370 (0.004)** | 0.162 (0.318) | **0.353**  **(0.020)**^a^ | 0.045 (0.772)^a^ | 0.195  (0.301)^a^ |
| **Total body fat (%)** | **0.910 (<0.001)** | **0.907 (<0.001)** | **0.830 (<0.001)** | **0.430 (0.001)** | **0.753 (<0.001)** | **0.511 (0.001)** | 0.208 (0.165)^a^ | 0.017 (0.910)^a^ | 0.337 (0.063)^a^ |
| **HOMA-IR** | **0.523 (<0.001)** | **0.578 (<0.001)** | **0.495 (<0.001)** | 0.143 (0.313) | **0.479 (<0.001)** | 0.167 (0.329) | **0.472 (0.001)**^a^ | -0.110 (0.499)^a^ | 0.092 (0.629)^a^ |
| **Mean systolic blood pressure** | -0.124 (0.269) | -0.094 (0.451) | -0.222 (0.097) | -0.089 (0.508) | -0.245 (0.060) | -0.054 (0.733) | -0.047 (0.755) ^a^ | 0.173 (0.256) ^a^ | -0.133 (0.475) ^a^ |
| **Mean diastolic blood pressure** | **0.373 (0.001)** | **0.529 (<0.001)** | 0.216 (0.106) | 0.260 (0.051) | 0.204 (0.118) | 0.228 (0.147) | 0.048 (0.747) ^a^ | 0.202 (0.183) ^a^ | -0.036 (0.846) ^a^ |
| **Waist circumference** | **0.278 (0.012)** | **0.260 (0.035)** | 0.021 (0.875) | 0.189 (0.160) | 0.185 (0.156) | 0.134 (0.398) | 0.074 (0.621) ^a^ | 0.118 (0.441) ^a^ | 0.058 (0.758) ^a^ |
| **HDL cholesterol** | 0.129 (0.248) | -0.167 (0.180) | 0.182 (0.178) | 0.038 (0.776) | -0.184 (0.159) | 0.135 (0.400) | -0.036 (0.810) ^a^ | -0.270 (0.073) ^a^ | -0.035 (0.852) ^a^ |

Data is Spearmans rank coefficient R (p-value) unless otherwise indicated. P-values <0.05 are bold.

a. Pearsons correlation coefficient

O-GDM: offspring of women with gestational diabetes; O-T1DM: offspring of women with type 1 diabetes; O-BP: offspring of women from the background population.

**Table S6: Correlations between adiponectin plasma levels, gene expression, DNA methylation and clinical variables by offspring group**

| **Adiponectin** | | | | | | | | | |
| --- | --- | --- | --- | --- | --- | --- | --- | --- | --- |
| **Offspring data** | **Adiponectin plasma levels** | | | ***ADIPOQ* gene expression** | | | **Average *ADIPOQ* DNA methylation** | | |
|  | **O-GDM** | **O-T1DM** | **O-BP** | **O-GDM** | **O-T1DM** | **O-BP** | **O-GDM** | **O-T1DM** | **O-BP** |
| **Plasma levels** | - | - | - | 0.244 (0.062) | -0.005 (0.97) | **0.463 (0.002)** | -0.105 (0.503) | 0.018 (0.908) | **-0.426 (0.027)** |
| **Gene expression** | 0.244 (0.062) | -0.005 (0.97) | **0.463 (0.002)** | - | - | - | -0.109 (0.590) | **-0.352 (0.033)** | -0.033 (0.883) |
| **Average DNA methylation** | -0.105 (0.503) | 0.018 (0.908) | **-0.426 (0.027)** | -0.109 (0.590) | **-0.352 (0.033)** | -0.033 (0.883) | - | - | - |
| **BMI (kg/m^2^)** | -0.076 (0.500) | -0.189 (0.126) | -**0.342 (0.010)** | **-0.337 (0.009)** | **-0.455 (<0.001)** | **-0.470 (0.002)** | **0.402 (0.008)**^a^ | **0.382 (0.012)**^a^ | 0.168 (0.393)^a^ |
| **Fasting insulin** | **-0.305 (0.008)** | -0.241 (0.059) | **-0.263 (0.068)** | **-0.289 (0.036)** | **-0.380 (0.004)** | **-0.471 (0.003)** | **0.481 (0.002)**^a^ | 0.255 (0.117)^a^ | 0.142 (0.490)^a^ |
| **Fasting plasma glucose** | -0.011 (0.921) | -0.120 (0.332) | **-0.413 (0.002)** | -0.191 (0.147) | -0.079 (0.550) | -0.171 (0.285) | 0.215 (0.166)^a^ | 0.068 (0.665)^a^ | 0.368 (0.054)^a^ |
| **120-min plasma glucose** | 0.003 (0.977) | -0.061 (0.627) | **-0.381 (0.004)** | -0.076 (0.590) | 0.074 (0.580) | -0.215 (0.184) | **0.336**  **(0.034)**^a^ | -0.117 (0.461)^a^ | **0.411**  **(0.033)^a^** |
| **Total body fat (%)** | 0.004 (0.972) | -0.093 (0.456) | -0.141 (0.299) | -0.080 (0.551) | -0.135 (0.303) | **-0.312 (0.044)** | **0.349 (0.023)**^a^ | 0.166 (0.287)^a^ | 0.042 (0.832)^a^ |
| **HOMA-IR** | **-0.288 (0.013)** | **-0.247 (0.052)** | **-0.336 (0.020)** | **-0.321 (0.019)** | **-0.321 (0.016)** | **-0.573 (<0.001)** | **0.506 (0.001)**^a^ | 0.247 (0.129)^a^ | 0.207 (0.310)^a^ |
| **Mean systolic blood pressure** | -0.157 (0.162) | -0.155 (0.211) | -0.256 (0.057) | -0.232 (0.080) | **-0.363 (0.004)** | **-0.339 (0.028)** | 0.015 (0.925) ^a^ | **0.385 (0.011)** ^a^ | 0.100 (0.613) ^a^ |
| **Mean diastolic blood pressure** | -0.053 (0.640) | -0.073 (0.554) | **-0.469 (<0.001)** | -0.167 (0.209) | **-0.410 (0.001)** | **-0.426 (0.005)** | 0.093 (0.553) ^a^ | 0.193 (0.216) ^a^ | 0.097 (0.622) |
| **Waist circumference** | -0.180 (0.108) | -0.158 (0.202) | **-0.455 (<0.001)** | **-0.314 (0.016)** | **-0.424 (0.001)** | **-0.552 (<0.001)** | **0.420 (0.005)** ^a^ | **0.529 (<0.001)** ^a^ | 0.278  (0.152) ^a^ |
| **HDL cholesterol** | **0.339 (0.002)** | 0.048 (0.702) | **0.391 (0.003)** | **0.286 (0.028)** | 0.215 (0.100) | **0.536 (<0.001)** | -0.190 (0.222) ^a^ | **-0.373 (0.014)** ^a^ | -0.400  (0.035) ^a^ |

Data is Spearmans rank coefficient R (p-value) unless otherwise indicated. P-values <0.05 are bold.

a. Pearsons correlation coefficient

O-GDM: offspring of women with gestational diabetes; O-T1DM: offspring of women with type 1 diabetes; O-BP: offspring of women from the background population.

**Table S7: Correlations between resistin plasma levels, gene expression, DNA methylation and clinical variables by offspring group**

| **Resistin** | | | | | | | | | |
| --- | --- | --- | --- | --- | --- | --- | --- | --- | --- |
| **Offspring data** | **Resistin plasma levels** | | | ***RETN* gene expression** | | | **Average *RETN* DNA methylation** | | |
|  | **O-GDM** | **O-T1DM** | **O-BP** | **O-GDM** | **O-T1DM** | **O-BP** | **O-GDM** | **O-T1DM** | **O-BP** |
| **Plasma levels** | - | - | - | -0.092 (0.487) | 0.143 (0.276) | 0.279 (0.073) | -0.205 (0.146) | -0.023 (0.878) | -0.303 (0.072) |
| **Gene expression** | -0.092 (0.487) | 0.143 (0.276) | 0.279 (0.073) | - | - | - | -0.011 (0.949) | 0.208 (0.192) | -0.039 (0.839) |
| **Average DNA methylation** | -0.205 (0.146) | -0.023 (0.878) | -0.303 (0.072) | -0.011 (0.949) | 0.208 (0.192) | -0.039 (0.839) | - | - | - |
| **BMI (kg/m^2^)** | **0.248 (0.024)** | 0.141 (0.254) | 0.091 (0.503) | -0.103 (0.436) | -0.084 (0.525) | -0.139 (0.378) | **-0.389 (0.004)**^a^ | -0.261 (0.076)^a^ | -0.102 (0.555)^a^ |
| **Fasting insulin** | 0.065 (0.584) | **0.307 (0.015)** | 0.258 (0.071) | -0.015 (0.717) | 0.011 (0.937) | 0.116 (0.494) | **-0.419 (0.003)**^a^ | **-0.302 (0.049)^a^** | -0.219 (0.220)^a^ |
| **Fastingplasma glucose** | 0.151 (0.177) | 0.215 (0.080) | 0.036 (0.792) | -0.162 (0.220) | **0.307 (0.015)** | 0.050 (0.757) | -0.218 (0.120)^a^ | -0.231 (0.118) | 0.014 (0.937)^a^ |
| **120-min plasma glucose** | 0.012 (0.918) | -0.003 (0.980) | 0.019 (0.891) | -0.091 (0.516) | 0.180 (0.173) | 0.098 (0.546) | -0.160 (0.278)^a^ | 0.019 (0.902)^a^ | -0.089 (0.611)^a^ |
| **Total body fat (%)** | **0.247 (0.026)** | 0.216 (0.079) | 0.175 (0.192) | -0.133 (0.319) | -0.058 (0.659) | 0.254 (0.105) | **-0.294 (0.036)^a^** | **-0.375 (0.009)**^a^ | **-0.359 (0.032)**^a^ |
| **HOMA-IR** | 0.109 (0.354) | **0.301 (0.018)** | **0.304 (0.033)** | -0.068 (0.628) | 0.038 (0.782) | 0.111 (0.519) | **-0.428 (0.002)**^a^ | **-0.326 (0.033)^a^** | -0.219 (0.221)^a^ |
| **Mean systolic blood pressure** | 0.089 (0.428) | 0.041 (0.744) | 0.038 (0.781) | -0.052 (0.696) | -0.088 (0.504) | **-0.308 (0.047)** | 0.036 (0.802)^a^ | -0.032 (0.830) ^a^ | -0.131 (0.448) ^a^ |
| **Mean diastolic blood pressure** | **0.314 (0.004)** | 0.173 (0.161) | 0.163 (0.227) | -0.069 (0.609) | -0.190 (0.146) | -0.290 (0.063) | -0.054 (0.704) ^a^ | -0.274 (0.062) ^a^ | **-0.367 (0.028)** ^a^ |
| **Waist circumference** | 0.084 (0.457) | 0.070 (0.573) | 0.007 (0.961) | -0.056 (0.675) | -0.065 (0.620) | -0.239 (0.127) | **-0.351 (0.011)** ^a^ | **-0.296 (0.043)** ^a^ | 0.034 (0.843) ^a^ |
| **HDL cholesterol** | 0.040 (0.721) | -0.188 (0.127) | 0.038 (0.778) | -0.017 (0.900) | -0.050 (0.703) | 0.251 (0.114) | 0.184 (0.193) ^a^ | 0.011 (0.941) ^a^ | 0.069 (0.689) ^a^ |

Data is Spearmans rank coefficient R (p-value) unless otherwise indicated. P-values <0.05 are bold.

a. Pearsons correlation coefficient

O-GDM: offspring of women with gestational diabetes; O-T1DM: offspring of women with type 1 diabetes; O-BP: offspring of women from the background population.
